# Astrocytic BDNF Modulates Sensitivity to Stress-Induced Anxiety-Like Behaviors

**DOI:** 10.34133/research.0818

**Published:** 2025-08-07

**Authors:** Wei-Peng Li, Gui-Yu Liu, Shi-Yun Wang, Jiao Hu, Wen-Juan Ji, Jun-Ming Zhu, Qing-Yu Chang, Tian-Yi Li, Guo-Rong Wei, Jian-Qing Shang, Hong-Zhan Li, Fu-Hua Peng, Yun-Yan Zhao, Xiao-Hong Su, Wei Xie

**Affiliations:** ^1^ Department of Neurology, Southern Medical University Hospital of Integrated Traditional Chinese and Western Medicine, Southern Medical University, Guangzhou 510317, China.; ^2^ School of Traditional Chinese Medicine, Southern Medical University, Guangzhou 510515, China.; ^3^ Guangdong Basic Research Center of Excellence for Integrated Traditional and Western Medicine for Qingzhi Diseases, Guangzhou 510515, China.; ^4^ Department of Neurology, The Third Affiliated Hospital of Sun Yat-Sen University, Guangzhou 510630, China.; ^5^ Department of Critical Care Medicine, The Affiliated Traditional Chinese Medicine Hospital of Guangzhou Medical University, Guangzhou 510140, China.; ^6^ Department of Traditional Chinese Medicine, Nanfang Hospital, Southern Medical University, Guangzhou 510515, China.

## Abstract

Chronic stress exposure is a potent risk factor for anxiety, disrupting adaptive responses and increasing vulnerability. Astrocytes, as essential regulators of synaptic function and neuroimmune homeostasis, are implicated in mood regulation and neuropsychiatric pathogenesis. However, the precise molecular mechanisms of astrocytes involved in stress-induced anxiety remain poorly understood. In this study, we reveal a pivotal role of astrocytic brain-derived neurotrophic factor (BDNF) in modulating anxiety sensitivity through coordinated regulation with hippocampal CA1 neurons. Chronic restraint stress induces anxiety-like behaviors and disrupts presynaptic glutamatergic transmission in hippocampal CA1 neurons, with astrocytes potentially playing a central regulatory role in a Ca^2+^-dependent manner. Pharmacological manipulation confirms the involvement of BDNF/TrkB signaling, while knockdown of astrocytic BDNF further impairs synaptic function and exacerbates stress-induced anxiety. Transcriptomic analysis suggests interferon-related signaling pathways as potential downstream effectors, amplifying anxiety sensitivity through altered astrocytic activation and neuroimmune dynamics. Our study provides critical mechanistic insights into astrocytic regulation of anxiety sensitivity and highlights astrocytic BDNF as a promising therapeutic target for stress-related anxiety disorders.

## Introduction

Anxiety disorders are highly prevalent mental diseases affecting nearly 300 million people worldwide and are characterized by excessive worry, fear, and uneasiness [1,2]. Stress serves as a major precipitating factor in the onset and exacerbation of anxiety, as prolonged stress exposure disrupts normal adaptive responses, heightening vulnerability to anxiety [3–6]. Despite evidence linking chronic stress to heightened anxiety sensitivity, the precise underlying mechanisms remain poorly understood.

As an essential component of the central nervous system, astrocytes are increasingly recognized for their important roles in maintaining brain function and homeostasis [7–9]. Beyond their traditional role in supporting neuronal functions, astrocytes are also involved in the processing of synaptic transmission and plasticity, thereby participating in mechanisms involved in cognition, memory, and mood regulation [8,10,11]. Additionally, astrocytes also produce a variety of neurotrophins and gliotransmitters, which are not only vital for physiological processes but also implicated in neuropsychiatric disorders, including anxiety [7,10–12].

Brain-derived neurotrophic factor (BDNF), a key neurotrophin expressed by astrocytes, plays an essential role in neuronal development and synaptic plasticity [13–16]. Alterations in BDNF signaling pathways have been associated with anxiety-like behaviors and the development of anxiety disorders [6,14,16,17]. While neuronal BDNF has been extensively studied, the role of astrocytic BDNF in modulating stress-induced anxiety sensitivity remains unclear. Specifically, how astrocytic BDNF influences neuronal activity and contributes to heightened anxiety following chronic stress warrants further investigation.

Here, we investigate how astrocytic BDNF modulates anxiety susceptibility. Our data reveal that chronic restraint stress (CRS) induces anxiety-like behaviors and diminishes presynaptic glutamatergic transmission in the hippocampal CA1. Importantly, astrocytic calcium activity contributes to heightened anxiety sensitivity, particularly when astrocyte silencing is combined with suppression of CA1 pyramidal neuron excitability. The BDNF/TrkB signaling plays a key role in this process, as pharmacological manipulation effectively modulates both electrophysiological and behavioral changes. Furthermore, the knockdown of astrocytic BDNF exacerbated anxiety sensitivity and related synaptic abnormalities, potentially through negative regulation of the downstream interferon (IFN) signaling pathway. Our findings underscore the crucial role of astrocytic BDNF in anxiety sensitivity, providing insights into potential therapeutic targets for anxiety disorders.

## Results

### CRS induces anxiety-like behaviors and alters presynaptic activity in hippocampal CA1

**Fig. 1.**
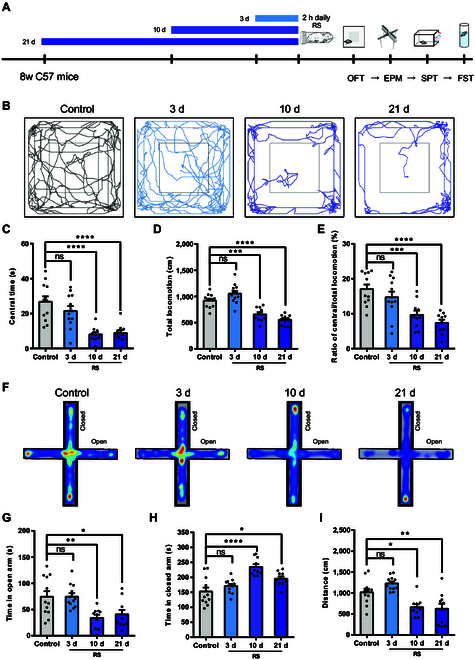
Restraint stress alters anxiety-like behaviors in mice (A) Timeline of behavioral experiments in mice subjected to restraint stress. (B) Representative movement tracks in the OFT. (C to E) Time in the center, total distance, and central/total locomotion ratio in the OFT [control, *n* = 12; 3d-RS, *n* = 12; 10d-RS, *n* = 10; 21d-RS, *n* = 10; one-way ANOVA; central time, *F*_(3, 40)_ = 14.10, *P* < 0.0001; total locomotion, *F*_(3, 40)_ = 29.49, *P* < 0.0001; central/total ratio, *F*_(3, 40)_ = 11.28, *P* < 0.0001]. (F) Representative heatmaps of time spent in EPM. (G to I) The time spent in open and closed arms, and distance traveled in the EPM [control, *n* = 12; 3d-RS, *n* = 12; 10d-RS, *n* = 10; 21d-RS, *n* = 10; one-way ANOVA; open arm, *F*_(3, 40)_ = 6.526, *P* = 0.0011; closed arm, *F*_(3, 40)_ = 13.06, *P* < 0.0001; distance, *F*_(3, 40)_ = 12.51, *P* < 0.0001].

It has been reported that restraint stress (RS) is a well-established rodent model for inducing both anxiety- and depression-like behaviors [18–21]. To develop a stable and reliable anxiety model, mice were subjected to 2 h of RS daily for different durations (3, 10, and 21 consecutive days) before conducting emotion-related behavioral assessments (Fig. 1A). Anxiety-like behaviors were assessed using the open field test (OFT) and elevated plus maze (EPM). Our results showed that RS for 10 and 21 consecutive days, but not 3 days, significantly reduced the central time in OFT (Fig. 1B and C), as well as decreased open arms time while increasing closed arms time in EPM (Fig. 1F to H), indicating successful induction of anxiety-like behaviors. Additionally, we noted a significant decrease in motor ability after 10 and 21 days of CRS (Fig. 1D and I). To rule out the influence of motor deficits on anxiety-like behaviors, we calculated the ratio of central to total distance traveled, which similarly decreased (Fig. 1E). Given the high comorbidity of anxiety and depression [22], we evaluated anhedonia and behavioral despair through sucrose preference test (SPT) and forced swim test (FST). Our data indicated that RS for 21 consecutive days, but not for 3 or 10 days, resulted in depressive-like behaviors (Fig. S1). These findings suggest that 10 days of CRS (referred to as the ordinary CRS protocol in subsequent experiments) effectively induces anxiety-like behaviors, with a positive correlation between CRS duration and anxiety sensitivity, while prolonged exposure (21 days) leads to depressive symptoms in mice.

**Fig. 2.**
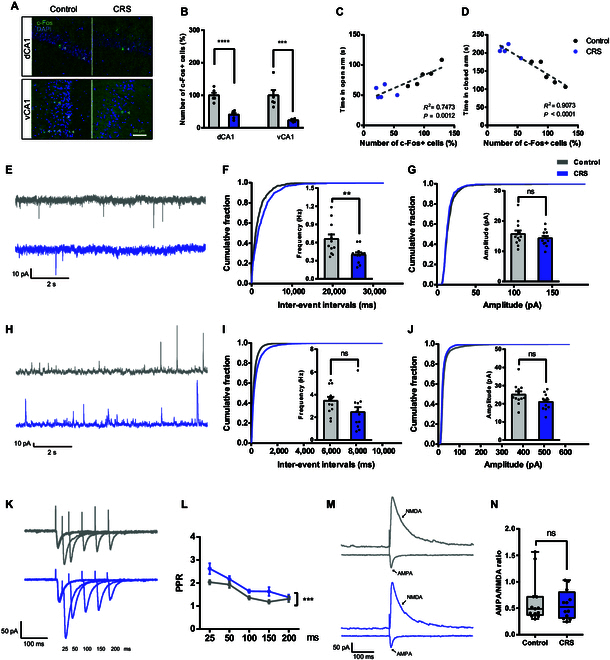
CRS alters hippocampal CA1 pyramidal neuronal activity. (A and B) Representative images and quantification of c-Fos^+^ cells in dorsal and ventral hippocampal CA1 regions from control and CRS-exposed groups (*n* = 6 per group; unpaired 2-tailed Student’s *t* test; dorsal CA1, *t*_10_ = 6.684, *P* < 0.0001; ventral CA1, *t*_10_ = 4.899, *P* = 0.0006). Scale bar, 50 μm. (C and D) Correlation between c-Fos^+^ cell count in the hippocampal CA1 region and time in open/closed arm (*n* = 5 per group; open arm, *P* = 0.0012, *R*^2^ = 0.7473; closed arm, *P* < 0.0001, *R*^2^ = 0.9073). (E) Example traces of 10-s recordings of sEPSCs. Scale bars, 10 pA, 2 s. (F and G) Cumulative probability plots of the sEPSCs inter-event intervals (IEIs) and amplitude, with insets depicting summary graphs of the frequency and amplitude of sEPSCs recorded from CA1 pyramidal neurons in control and CRS mice, respectively (*n* = 4 mice per group, average of 2 to 4 cells from each mouse; unpaired 2-tailed Student’s *t* test; frequency, *t*_22_ = 3.051, *P* = 0.0059; amplitude, *t*_22_ = 1.019, *P* = 0.3192). (H) Example traces of 10-s recordings of sIPSCs. Scale bars, 10 pA, 2 s. (I and J) Cumulative probability plots of the sIPSCs IEIs and amplitude, with insets depicting summary graphs of the frequency and amplitude of sIPSCs recorded from CA1 pyramidal neurons in control and CRS mice, respectively (*n* = 4 mice per group, average of 2 to 4 cells from each mouse; unpaired 2-tailed Student’s *t* test; frequency, *t*_22_ = 1.815, *P* = 0.0831; amplitude, *t*_22_ = 1.925, *P* = 0.0672). (K) Example traces of PPR recorded from CA1 pyramidal neurons in control and CRS mice. Scale bars, 50 pA, 100 ms. (L) Quantification of PPR [*n* = 4 mice per group, average of 2 to 4 cells from each mouse; 2-way ANOVA; *F*_(1, 115)_ = 14.75, *P* = 0.0002]. (M) Example traces of AMPA and NMDA current recorded from CA1 pyramidal neurons in control and CRS mice. Scale bars, 50 pA, 100 ms. (N) Quantification of AMPA/NMDA ratio recorded from CA1 pyramidal neurons in control and CRS mice (*n* = 4 mice per group, average of 2 to 4 cells from each mouse; Mann–Whitney test; *Z* = −0.0418, *P* = 0.9205).

To investigate the contribution of specific brain regions to CRS-induced anxiety, we assessed neuronal activity using c-Fos, a well-established marker of immediate early gene expression [23]. Significant alterations in c-Fos^+^ cell density were predominantly observed in the hippocampus (both dorsal and ventral subregions), whereas minimal changes were detected in other implicated regions (Fig. S2A and B). Within the hippocampus, CRS significantly reduced the number of c-Fos^+^ cells in the CA1 region, including dorsal and ventral CA1, while no significant changes were observed in other subregions (Fig. 2A and B and Fig. S2C and D). Moreover, c-Fos expression in CA1 positively correlated with time spent in the open arms and negatively correlated with time in the closed arms of the EPM, suggesting a functional link between CA1 activity and anxiety-related behavior (Fig. 2C and D).

To further assess synaptic function, we performed whole-cell patch-clamp recordings in CA1 pyramidal neurons. CRS significantly reduced the frequency, but not the amplitude, of spontaneous excitatory postsynaptic currents (sEPSCs) (Fig. 2E to G), while the spontaneous inhibitory postsynaptic currents (sIPSCs) showed no significant changes (Fig. 2H to J). Consistently, the paired-pulse ratio (PPR) increased significantly in CRS mice (Fig. 2K and L). The α-amino-3-hydroxy-5-methyl-4-isoxazolepropionic acid (AMPA) to N-methyl-D-aspartate (NMDA) current ratio remained unchanged (Fig. 2M and N). No significant alterations were observed in sEPSCs and sIPSCs frequency or amplitude in dentate granule cells (Fig. S2E to J). Together, these results suggest a decreased presynaptic glutamate release probability, possibly leading to reduced activity of hippocampal CA1 neurons in CRS-induced anxiety-like behaviors.

To further explore this relationship, we employed inhibitory DREADD (hM4Di) to selectively suppress CA1 pyramidal neuron activity via AAV-CaMK2α-hM4Di-mCherry during the anxiety behavioral tests. Unexpectedly, this inhibition did not directly induce anxiety-like behaviors (Fig. S3), suggesting that while the reduction in presynaptic glutamate release in CA1 neurons may be associated with increased sensitivity to anxiety-like behaviors, it is not sufficiently a direct causative factor.

### Astrocytes modulate CRS-induced anxiety-like behaviors

**Fig. 3.**
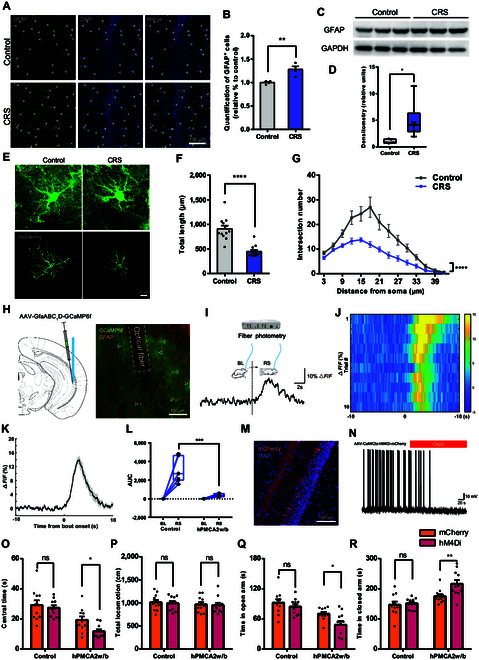
Astrocytes modulate CRS-induced anxiety-like behaviors in collaboration with CA1 pyramidal neuron. (A and B) Representative images and quantification of GFAP^+^ astrocytes in CA1 (*n* = 4 mice per group; unpaired 2-tailed Student’s *t* test; *t*_6_ = 4.271, *P* = 0.0053). Scale bar, 100 μm. (C and D) Western blotting analysis of GFAP protein levels (*n* = 6 mice per group; Mann–Whitney test; *Z* = 3.076, *P* = 0.0022). (E to G) 3D reconstruction and quantification of AAV-GFAP-eGFP-labeled astrocytes [*n* = 12 eGFP^+^ astrocytes from 4 mice per group; total length, unpaired 2-tailed Student’s *t* test; *t*_22_ = 6.176, *P* < 0.0001; Sholl, 2-way ANOVA, *F*_(1, 308)_ = 97.29, *P* < 0.0001]. Scale bar, 10 μm. (H) Left: Schematic illustrating AAV-GfaABC_1_D-GCaMP6f injection and optical fiber implantation. Right: Representative images showing viral expression in CA1 astrocytes. Scale bar, 100 μm. (I) Representative Ca^2+^ signal trace in CA1 astrocytes in response to restraint stress stimulation. Scale bars, 10% Δ*F*/*F*, 2 s. (J and K) Representative heatmaps and averaged Ca^2+^ transients evoked by restraint stress stimulation. (L) Quantification of area under the curve (AUC) of Ca^2+^ signals at baseline (BL) and during restraint stress in control and hPMCA2w/b mice [control, *n* = 7; hPMCA2w/b, *n* = 4; 2-way ANOVA; *F*_(1, 18)_ = 15.05, *P* = 0.0011]. (M) Representative CA1 images showing hM4Di-mCherry expression. Scale bar, 100 μm. (N) Representative current-clamp recording of hM4Di-expressing CA1 pyramidal neuron with CNO application. Scale bars, 10 mV, 20 s. (O and P) Center time and locomotion in OFT in control and hPMCA2w/b mice after 3-day subthreshold CRS with hM4Di treatment [*n* = 11 mice per group; 2-way ANOVA; central time, *F*_(1, 40)_ = 4.335, *P* = 0.0438; total locomotion, *F*_(1, 40)_ = 0.1386, *P* = 0.7116]. (Q and R) Time spent in the open and closed arms of the EPM in control and hPMCA2w/b mice after 3-day subthreshold CRS with hM4Di treatment [*n* = 11 mice per group; 2-way ANOVA; open arm, *F*_(1, 40)_ = 6.013, *P* = 0.0187; closed arm, *F*_(1, 40)_ = 5.831, *P* = 0.0204].

Emerging evidence reveals that astrocytes play an integral role in synaptic function and mood regulation [10,11]. To explore the role of astrocytes in modulating CRS-induced anxiety-like behaviors, we performed staining for glial fibrillary acidic protein (GFAP), an astrocyte marker, to assess for astrocyte activation. CRS mice exhibited a significant increase in GFAP^+^ cells (Fig. 3A and B), corroborated by Western blot analysis showing elevated GFAP protein expression (Fig. 3C and D). Utilizing AAV-GFAP-eGFP for sparse labeling, we observed notable morphological changes in hippocampal astrocytes after CRS, characterized by reduced total process length and decreased branching complexity, as assessed by Sholl analysis (Fig. 3E to G).

To further evaluate the role of astrocytes in stress-induced anxiety-like behaviors, we employed fiber photometry to directly record astrocytic calcium responses to the RS in freely moving mice. Following injection of AAV-GfaABC_1_D-GCaMP6f into the ventral CA1 region of the hippocampus, GCaMP6f was specifically expressed in astrocytes (Fig. 3H). An optical fiber was then implanted above the ventral CA1 to record fluorescence changes, and we observed a significant increase in astrocytic Ca^2+^ activity in response to RS stimulation (Fig. 3I to L).

To examine whether concurrent perturbation of astrocytes and CA1 pyramidal neurons influences stress vulnerability, we employed a dual-manipulation strategy in subthreshold CRS model mice. We selectively inhibited CA1 pyramidal neuron activity using hM4Di DREADDs, as well as attenuated astrocytic calcium signaling by expressing the human plasma membrane Ca^2+^ ATPase isoform 2w/b (hPMCA2w/b) [24], under the control of the astrocyte-specific GfaABC_1_D promoter (Fig. 3L to N and Fig. S4). In subthreshold CRS model mice, hM4Di inhibition aggravated anxiety-like behaviors in hPMCA2w/b-treated mice, without affecting motor abilities across groups (Fig. 3O to R). Taken together, these results highlight the crucial role of astrocytes in modulating sensitivity to CRS-induced anxiety-like behaviors through coordinated regulation with CA1 pyramidal neurons.

### The BDNF-TrkB signaling contribute to the alterations of anxiety susceptibility and excitatory synaptic transmission

**Fig. 4.**
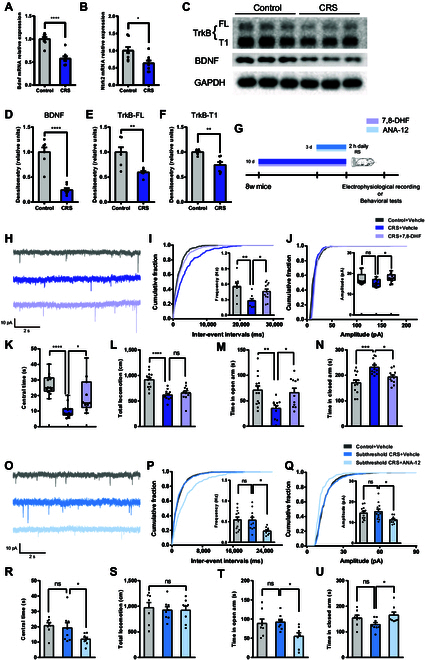
BDNF-TrkB pathway modulates excitatory synaptic transmission and anxiety susceptibility. (A and B) RT-qPCR analysis of *Bdnf* and *Ntrk2* in hippocampus from control and CRS mice (*n* = 8 mice per group; unpaired 2-tailed Student’s *t* test; *Bdnf*, *t*_14_ = 6.389, *P* < 0.0001; *Ntrk2*, *t*_14_ = 2.795, *P* = 0.0143). (C to F) Western blotting analysis of BDNF and TrkB (FL and T1 isoforms) protein (*n* = 7 mice per group; unpaired 2-tailed Student’s *t* test; BDNF, *t*_12_ = 7.444, *P* < 0.0001; TrkB-FL, *t*_12_ = 4.251, *P* = 0.0011; TrkB-T1, *t*_12_ = 3.936, *P* = 0.002). (G) Experimental timeline for behavioral assays in mice treated with 7,8-DHF or ANA-12 under CRS or subthreshold CRS protocols, respectively. (H) Example traces of 10-s recordings of sEPSCs from CA1 pyramidal neurons in control + vehicle, CRS + vehicle, and CRS + 7,8-DHF mice, as indicated. Scale bars, 10 pA, 2 s. (I and J) Cumulative probability plots of the sEPSCs inter-event intervals (IEIs) and amplitude, with insets depicting summary graphs of the frequency and amplitude [control + vehicle, *n* = 3 mice; CRS + vehicle, *n* = 3 mice; CRS + 7,8-DHF, *n* = 4 mice; average of 2 to 3 cells from each mouse; frequency, one-way ANOVA, *F*_(2, 26)_ = 6.449, *P* = 0.0053; amplitude, Kruskal–Wallis test, *P* = 0.0461]. (K and L) Center time and locomotion in the OFT for 7,8-DHF treatment [control + vehicle, *n* = 13; CRS + vehicle, *n* = 11; CRS + 7,8-DHF, *n* = 11; central time, Kruskal–Wallis test, *P* < 0.0001; total locomotion, one-way ANOVA, *F*_(2, 32)_ = 17.03, *P* < 0.0001]. (M and N) Open and closed arm time in EPM [control + vehicle, *n* = 13; CRS + vehicle, *n* = 11; CRS + 7,8-DHF, *n* = 11; one-way ANOVA; open arm, *F*_(2, 32)_ = 5.991, *P* = 0.0062; closed arm, *F*_(2, 32)_ = 9.975, *P* = 0.0004]. (O) Example traces of 10-s recordings of sEPSCs from CA1 pyramidal neurons in control + vehicle, subthreshold CRS + vehicle, and subthreshold CRS + ANA-12 mice, as indicated. Scale bars, 10 pA, 2 s. (P and Q) Cumulative probability plots of the sEPSCs IEIs and amplitude, with insets depicting summary graphs of the frequency and amplitude [*n* = 4 mice per group, average of 2 to 4 cells from each mouse; one-way ANOVA; frequency, *F*_(2, 28)_ = 4.549, *P* = 0.0195; amplitude, *F*_(2, 28)_ = 3.615, *P* = 0.0401]. (R and S) Central time and locomotion in OFT for ANA-12 treatment [*n* = 8 per group; one-way ANOVA; central time, *F*_(2, 21)_ = 4.070, *P* = 0.0321; total locomotion, *F*_(2, 21)_ = 0.1169, *P* = 0.8903]. (T and U) Open and closed arm time in EPM [*n* = 8 per group; one-way ANOVA; open arm, *F*_(2, 21)_ = 4.699, *P* = 0.0206; closed arm, *F*_(2, 21)_ = 3.649, *P* = 0.0436].

Previous studies have demonstrated that reductions in astrocytic Ca^2+^ levels impair BDNF release from astrocytes [25]. BDNF-TrkB signaling has been implicated in the regulation of stress responses and negative emotion such as anxiety [14,26,27]. To investigate the involvement of this pathway in CRS-induced anxiety, we examined hippocampal BDNF and TrkB expression using quantitative PCR and Western blot analysis. CRS-exposed mice exhibited significantly reduced BDNF and TrkB mRNA and protein levels (Fig. 4A to F), indicating a potential role of impaired BDNF-TrkB signaling in the development of anxiety-like behaviors.

To directly evaluate its functional role in anxiety susceptibility, we employed pharmacological interventions (Fig. 4G). Our results showed that administration of 7,8-dihydroxyflavone (7,8-DHF), a BDNF mimetic, effectively restored sEPSCs frequency and significantly increased sEPSCs amplitude in CRS mice (Fig. 4H to J). Behavioral results indicated that 7,8-DHF treatment alleviated anxiety-like behaviors, including increased central time in the OFT and open-arm time while decreasing closed-arm time in the EPM compared to CRS + vehicle mice (Fig. 4K to N). It is noteworthy that acute 7,8-DHF administration had no behavioral effects in the absence of CRS (Fig. S5). Conversely, treatment with ANA-12, a specific BDNF-TrkB inhibitor, significantly decreased in both sEPSCs frequency and amplitude in the subthreshold CRS mice (Fig. 4O to Q). Behaviorally, ANA-12-treated mice exhibited exacerbated anxiety-like behaviors, with decreased central time in the OFT and reduced open-arm time in the EPM, without affecting locomotion (Fig. 4R to U).

These results indicate BDNF-TrkB signaling as a critical mediator of stress-induced anxiety susceptibility, linking alterations in excitatory synaptic transmission to anxiety-like behaviors.

### Astrocytic BDNF in hippocampal CA1 regulates susceptibility to stress-induced anxiety-like behaviors

**Fig. 5.**
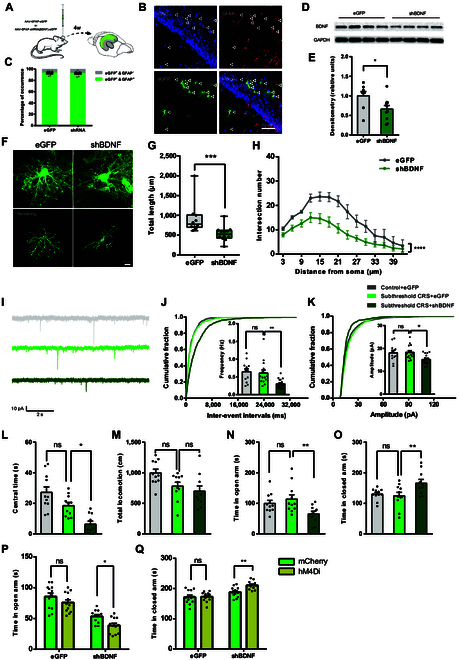
Astrocytic BDNF in CA1 regulates stress-induced anxiety-like behaviors. (A) Schematic of AAV-GFAP-shRNA (BDNF) injection strategy. (B) Representative images showing AAV distribution in CA1 with eGFP (green), GFAP (red), and DAPI (blue). Scale bar, 50 μm. (C) Colocalization quantification in AAV-GFAP-shBDNF-eGFP and its control AAV-GFAP-eGFP (eGFP, *n* = 8 slices from 4 mice; shBDNF, *n* = 10 slices from 4 mice). (D and E) Western blotting analysis of BDNF in eGFP- and shBDNF-treated CA1 hippocampus (eGFP, *n* = 8; shBDNF, *n* = 10; unpaired 2-tailed Student’s *t* test; *t*_16_ = 2.343, *P* = 0.0324). (F to H) 3D reconstructions and quantification of AAV-labeled astrocytes [eGFP, *n* = 10 astrocytes from 4 mice; shBDNF, *n* = 12 astrocytes from 4 mice; total length, Mann–Whitney test; *Z* = −280.7, *P* = 0.0003; Sholl, 2-way ANOVA, *F*_(1, 280)_ = 73.38, *P* < 0.0001]. Scale bar, 10 μm. (I) Example traces of 10-s recordings of sEPSCs from CA1 pyramidal neurons in shBDNF-eGFP and control eGFP with subthreshold CRS treatment. Scale bars, 10 pA, 2 s. (J and K) Cumulative probability plots of the sEPSCs inter-event intervals (IEIs) and amplitude, with insets depicting summary graphs of the frequency and amplitude of sEPSC [control + eGFP, *n* = 5 mice; subthreshold CRS + eGFP, *n* = 5 mice; subthreshold CRS + shBDNF, *n* = 6 mice; average of 2 to 4 cells from each mouse; one-way ANOVA ; frequency, *F*_(2, 41)_ = 7.987, *P* = 0.0012; amplitude, *F*_(2, 41)_ = 5.567, *P* = 0.0073]. (L and M) Center time and locomotion in OFT [control + eGFP, *n* = 11 mice; subthreshold CRS + eGFP, *n* = 11 mice; subthreshold CRS + shBDNF, *n* = 10 mice; one-way ANOVA; central time, *F*_(2, 29)_ = 14.98, *P* < 0.0001; total locomotion, *F*_(2, 29)_ = 4.877, *P* = 0.0149]. (N and O) Open and closed arm time in EPM [control + eGFP, *n* = 11 mice; subthreshold CRS + eGFP, *n* = 11 mice; subthreshold CRS + shBDNF, *n* = 10 mice; one-way ANOVA; open arm, *F*_(2, 29)_ = 4.719, *P* = 0.0168; closed arm, *F*_(2, 29)_ = 4.825, *P* = 0.0155]. (P and Q) EPM open/closed arm time in hM4Di-treated AAV-GFAP-shBDNF-eGFP mice under 3-day subthreshold CRS [*n* = 12 mice per group; 2-way ANOVA; open arm, *F*_(1, 44)_ = 7.836, *P* = 0.0076; closed arm, *F*_(1, 44)_ = 5.678, *P* = 0.0216].

To explore the specific role of astrocytic BDNF in anxiety modulation, we used AAV-mediated short hairpin RNA (shRNA) to knock down BDNF expression in hippocampal astrocytes. Mice were stereotaxically injected with AAV-GFAP-shRNA (BDNF)-eGFP (shBDNF) or control AAV-GFAP-eGFP, and sacrificed 4 weeks postinjection to allow for complete viral expression (Fig. 5A). Successful transduction was confirmed by colocalization of eGFP with GFAP immunostaining, indicating astrocyte-specific expression, as well as a significant reduction in BDNF protein expression in the shBDNF group (Fig. 5B to E).

Morphological analysis of astrocytes with BDNF knockdown revealed significant reductions in both total process length and branching complexity (Fig. 5F to H). Electrophysiologically, frequency and amplitude of sEPSCs, but not sIPSCs, in adjacent hippocampal CA1 neurons were significantly reduced in the subthreshold CRS + shBDNF group compared to the subthreshold CRS + eGFP group (Fig. 5I to K and Fig. S6A to C). Behaviorally, shBDNF mice subjected to subthreshold CRS exhibited exacerbated anxiety-like behaviors, as indicated by decreased central time in the OFT, reduced duration in the open arms, and increased duration in the closed arms in the EPM compared to the subthreshold CRS + eGFP group (Fig. 5L to O).

To further explore the astrocyte–neuron interplay, we employed hM4Di to selectively suppress CA1 pyramidal neuron activity in subthreshold CRS + shBDNF mice. DREADD-mediated inhibition aggravated anxiety-like behaviors in EPM (Fig. 5P and Q) and marginally increased anxiety-like behaviors in OFT without statistical significance (Fig. S6D). Locomotion was unaffected across all groups (Fig. S6E).

Collectively, these results reveal that astrocytic BDNF in hippocampal CA1 modulates the susceptibility to stress-induced anxiety-like behaviors, highlighting the importance of interactions between astrocytes and neurons in regulating anxiety sensitivity.

### Astrocytic BDNF regulates stress-induced anxiety susceptibility via the IFN signaling pathway

**Fig. 6.**
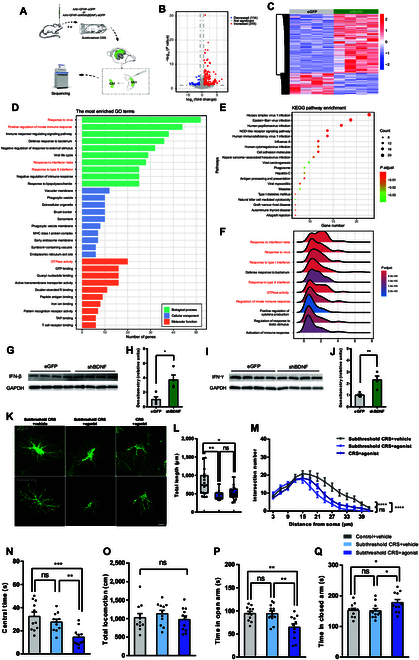
Astrocytic BDNF regulates anxiety sensitivity through interferon signaling. (A) Schematic for RNA-seq analysis. (B) Volcano plots illustrating differentially expressed genes (DEGs) in the CA1 region of shBDNF-eGFP and control eGFP mice (319 up-regulated and 114 down-regulated). (C) Heatmap of DEGs expression (red = high, blue = low). (D to F) GO, KEGG, and GSEA enrichment analyses. (G to J) Western blotting analysis of IFN-β and IFN-γ protein in the eGFP- and shBDNF-treated CA1 hippocampus (*n* = 4 mice per group; unpaired 2-tailed Student’s *t* test; IFN-β, *t*_6_ = 3.571, *P* = 0.0118; IFN-γ, *t*_6_ = 3.819, *P* = 0.0088). (K to M) 3D reconstructions and quantifications of astrocytes across IFN agonist-treated groups [subthreshold CRS + vehicle, *n* = 17 astrocytes from 4 mice; subthreshold CRS + agonist, *n* = 11 astrocytes from 4 mice; CRS + agonist, *n* = 16 astrocytes from 4 mice; total length, Kruskal–Wallis test, *P* = 0.0032; Sholl, 2-way ANOVA, *F*_(2, 574)_ = 44.64, *P* < 0.0001]. Scale bar, 10 μm. (N and O) Central time and total locomotion in the OFT [*n* = 11 mice per group; one-way ANOVA; central time, *F*_(2, 30)_ = 10.23, *P* = 0.0004; total locomotion, *F*_(2, 30)_ = 0.6330, *P* = 0.5379]. (P and Q) The open and closed arms time in the EPM [*n* = 11 mice per group; one-way ANOVA; open arm, *F*_(2, 30)_ = 7.377, *P* = 0.0025; closed arm, *F*_(2, 30)_ = 3.54, *P* = 0.0417].

To explore the downstream mechanisms by which astrocytic BDNF modulates sensitivity to stress-induced anxiety-like behaviors, we employed high-throughput transcriptome sequencing. Mice were stereotaxically injected with AAV-GFAP-shBDNF or AAV-GFAP-eGFP and 4 weeks later, subjected to a 3-day subthreshold CRS model. AAV-infected hippocampal CA1 tissues, identified by eGFP, were precisely dissected under a fluorescence microscope for transcriptome sequencing (Fig. 6A). Differential expression analysis revealed 319 up-regulated and 114 down-regulated genes between groups, visualized through volcano plot and heatmap (Fig. 6B and C). Gene Ontology (GO) and Kyoto Encyclopedia of Genes and Genomes (KEGG) analyses linked these genes primarily to innate immune responses and viral/bacterial defense, implicating IFN signaling pathways (Fig. 6D and E and Fig. S7). Gene set enrichment analysis (GSEA) further supported and highlighted the involvement of IFN-related pathways (Fig. 6F). Western blot analysis corroborated the transcriptomic results, showing significant up-regulation of IFN-β and IFN-γ protein expressions in shBDNF-treated mice as well as in CRS-exposed mice (Fig. 6G to J and Fig. S8A to D). Immunofluorescence analysis demonstrated increased IFN-β expression in both GFAP^+^ astrocytes and IBA1^+^ microglia, while IFN-γ was significantly elevated in astrocytes but not in microglia (Fig. S8E to H).

To determine the functional relevance of IFN signaling in anxiety susceptibility, mice were treated with tilorone dihydrochloride, an IFN inducer. Tilorone treatment significantly altered astrocyte morphology after 3 days of subthreshold CRS, as evidenced by reductions in total process length and branching complexity. However, no additional changes occurred after 10 days (Fig. 6K to M). Behaviorally, tilorone induced anxiety-like behaviors following 3 days of subthreshold CRS, as indicated by the decreased time spent in the center in the OFT, as well as the reduced time in the open arms and the increased time in the closed arms in the EPM (Fig. 6N to Q).

In summary, these results suggest that activation of the IFN signaling promotes stress-induced anxiety-like behaviors and may serve as a crucial downstream effector of astrocytic BDNF signaling.

## Discussion

**Fig. 7.**
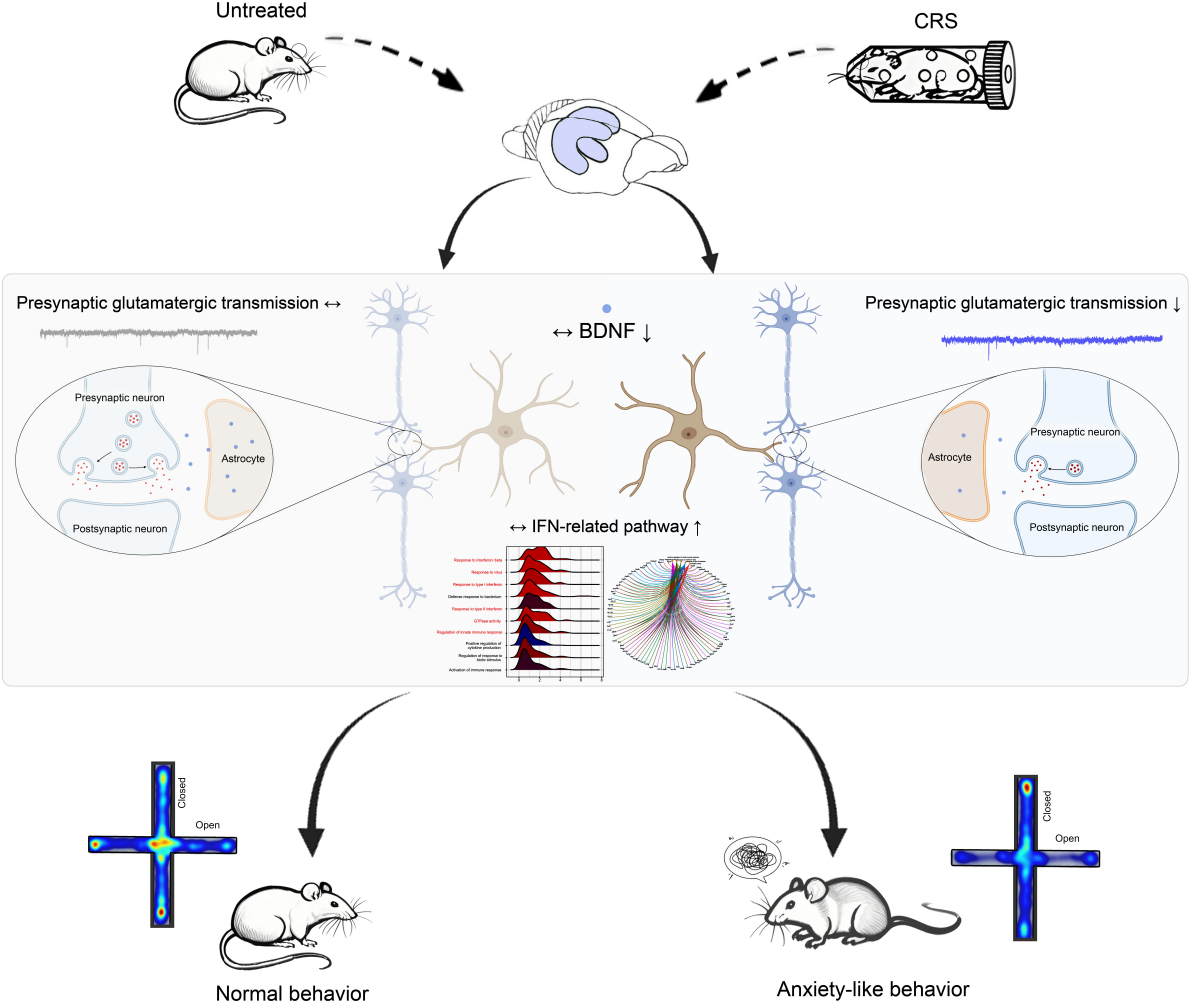
Proposed model. CRS reduces astrocytic BDNF expression, leading to disrupted presynaptic glutamatergic transmission in hippocampal CA1 and increased anxiety sensitivity. This dysregulation is further exacerbated by altered IFN-related signaling pathways, which modulate astrocytic activation and the neuroimmune environment, ultimately heightening susceptibility to stress-induced anxiety.

This study investigated the role of astrocytic BDNF in modulating susceptibility to stress-induced anxiety. Our results demonstrated that CRS alters both anxiety-like behaviors and presynaptic glutamatergic transmission in hippocampal CA1 neurons, with astrocytes playing a central regulatory role. The BDNF-TrkB signaling pathway emerged as a key mediator, as pharmacological interventions of this pathway affected electrophysiological and anxiety-like behavioral changes. Astrocytic BDNF knockdown exacerbated synaptic dysfunction and heightened anxiety sensitivity, suggesting its essential role in neuronal responses to stress. Our data further revealed the IFN signaling pathway as a potential downstream effector of astrocytic BDNF in regulating anxiety. Thus, these findings highlight the critical role of astrocytic BDNF in modulating anxiety sensitivity through coordinated regulation with CA1 pyramidal neurons, as shown in Fig. 7.

Chronic stress has been identified as a major risk factor in the development of anxiety disorders [3–6,28,29]. To establish a reliable model of anxiety-like behaviors, we employed CRS, a widely used stress paradigm in laboratory animals to simulate human psychological stress [5,18–21]. Our results found that CRS, a mild intervention that avoids direct physical harm to mice, effectively induced anxiety-like behaviors. As it has been controversially reported that CRS could induce both anxiety- and depression-like phenotypes [18–21,30], we systematically evaluated the impact of different CRS durations (3, 10, and 21 days) using various affective behavioral assays. Our findings revealed that 10 days of CRS was optimal for inducing robust anxiety-like behaviors, as indicated by reduced central time and central distance ratio in OFT, as well as decreased duration in the open arms and increased in the closed arm times in EPM. While 21 days of CRS also induced anxiety-like behaviors, it was accompanied by depressive-like symptoms, complicating the interpretation of anxiety outcomes. Conversely, 3 days of CRS was insufficient to induce noticeable anxiety- or depressive-like behaviors. Thus, our results indicate that 10 days of CRS provides a balanced model for studying anxiety-like behaviors without the confounding effects of depression. Accordingly, we adopted the 10-day protocol as the standard for anxiety modeling, while the 3-day CRS served as a complementary subthreshold model to explore mechanisms underlying stress sensitivity.

Hippocampus, as an important component in the limbic system, is essential for cognitive function and emotional regulation, including anxiety [31–33]. To investigate its involvement in CRS-induced anxiety-like behaviors, we examined multiple brain regions and hippocampal subregions, identifying CA1, but not the DG, as a key area involved, as evidenced by decreased c-Fos immunofluorescence. We further observed a decreased excitatory/inhibitory (E/I) balance in CA1 neurons through whole-cell patch-clamp recordings, suggesting diminished presynaptic excitability. This aligns with prior findings that weakened excitatory input in CA1 neurons contributes to anxiety-like behaviors [34,35]. However, chemogenetic inhibition of CA1 pyramidal neurons unexpectedly failed to induce anxiety-like behaviors, suggesting that CRS-induced anxiety is not solely driven by alterations in CA1 neuronal activity but rather by a more complex mechanism involving finely tuned interactions between neurons and other cell types, such as astrocytes. Supporting this notion, recent studies have showed that hippocampal astrocyte calcium activity bidirectionally modulates innate and stress-induced anxiety-like behaviors [12,36]. These findings indicate that collaborative regulation between astrocytes and neurons may contribute to more nuanced control of synaptic plasticity that extends beyond simplistic alterations in neuronal firing.

Recently, astrocytes have gained increasing attention for their diverse and essential roles in the CNS. Beyond their typical support functions, astrocytes actively participate in modulating synaptic transmission and maintain neurotransmitter homeostasis through the tripartite synapse [10,11,37,38]. Our results showed that CRS-modeled mice exhibited increased GFAP expression and notable morphological changes in astrocytes, indicative of an activated state in response to chronic stress. Astrocytes communicate with neurons and other glial cells through intracellular calcium signaling [36,38–40]. Our study demonstrated that hPMCA2w/b inhibition of Ca^2+^ signals in astrocytes led to an increased sensitivity of mice to CRS-induced anxiety-like behaviors. Moreover, when combining silencing of astrocytic calcium transients with chemogenetic inhibition of CA1 pyramidal neurons, we observed a pronounced exacerbation of anxiety-like behaviors, highlighting the crucial role of astrocytic regulation of neuronal excitability in modulating sensitivity to stress-induced anxiety. Although astrocytes were historically considered a homogeneous population, there is growing evidence that indicates that astrocytes exhibit morphological, molecular, and inter- and intra-subregional functional heterogeneity in the adult hippocampus; differentially respond to local neuronal activity; and regulate synaptic function [36,41,42]. Astrocytes communicate with neurons via calcium signaling, triggering the release of various active substances, such as BDNF, D-serine, and adenosine triphosphate (ATP), which modulate synaptic function [10,37].

BDNF and its receptor, TrkB, are well-documented modulators of synaptic function, neuronal network formation, and behaviors, including anxiety [14,26,27]. In our study, hippocampal BDNF/TrkB expression was significantly down-regulated in the CRS-exposed mice, and pharmacological modulation of this pathway influenced synaptic transmission and anxiety-like behaviors. These findings align with prior studies implicating BDNF in anxiety modulation, though most of the existing research has focused on general roles of BDNF rather than specific contributions to anxiety disorders. For instance, studies on BDNF^Val66Met^ polymorphism have shown that mice with the Met allele have impaired BDNF secretion and become more susceptible to stress-induced phenotypes, such as decreased sucrose preference in the SPT, increased immobility in the FST, and enhanced anxiety-like behaviors on the EPM [43]. These findings suggest that the Val66Met polymorphism contributes to trait anxiety independently of psychiatric diagnoses, though the etiologies of anxiety disorders are likely more complex and distinct [43–46]. Our results further support the critical role of BDNF in modulating susceptibility to stress-induced anxiety. Astrocytes are one of the major sources of BDNF in the CNS [47]. Using AAV-mediated astrocytic BDNF knockdown mice, we further demonstrated that astrocyte-derived BDNF is indispensable for regulating anxiety-like sensitivity through its impact on glutamatergic synaptic transmission. Combined with chemogenetic inhibition of CA1 pyramidal neurons, the knockdown manipulations exacerbated anxiety-like behaviors, emphasizing a synergistic effect between astrocytic BDNF signaling and neuronal activity in modulating stress response.

Interestingly, astrocytes displayed noticeable morphological changes under stress-induced anxiety or following knockdown of astrocytic BDNF, suggesting their potential heterogeneity based on morphology. Emerging research increasingly recognizes astrocytes as dynamic and heterogeneous modulators of brain function, capable of rapidly responding to external stimuli with diverse molecular and morphological adaptations [48–51]. To uncover the mechanisms underlying these alterations, we performed transcriptomic sequencing on CA1 tissue from stress-exposed mice with astrocytic BDNF knockdown, identifying significant involvement of the IFN signaling pathway. Our finding suggests that IFN signaling may act as a mediator between astrocytic BDNF and anxiety sensitivity. The role of IFN signaling in the CNS, particularly in regulating astrocytic function, has gained increasing attention in recent years [52–55]. Single-cell RNA sequencing studies have indicated that IFN-related pathways modulate astrocytic activation and contribute to neuroinflammatory responses [53,55]. However, little is known about how IFN signaling influences astrocyte-mediated regulation of anxiety sensitivity. Our data showed that astrocytic BDNF knockdown resulted in up-regulation of IFN signaling, possibly exacerbating anxiety sensitivity through altered astrocytic activity and a disrupted neuroimmune environment. These results suggest a potential feedback mechanism in which reduced BDNF levels elevate IFN signaling, sensitizing the brain’s response to stress and amplifying anxiety-like behaviors.

The present study has several limitations. First, the current pharmacological and viral strategies cannot completely exclude contributions from non-astrocytic sources of BDNF. To more precisely delineate the cell-type-specific contributions to stress vulnerability, studies employing BDNF/TrkB manipulations in other cell types (e.g., neuron or microglia) are needed. Second, the study did not perform astrocyte-specific BDNF overexpression experiments, which would provide critical complementary evidence to determine whether astrocytic BDNF directly mitigates stress-induced anxiety-like behaviors. Third, while our findings implicate astrocytic BDNF in modulating IFN pathway activation, the mechanistic hierarchy (i.e., upstream–downstream causality) remain to be elucidated. Cell type-specific manipulation of IFN signaling will be essential to clarify the direct interactions involved. Finally, the use of systemic pharmacological administration (intraperitoneally [i.p.]) limits anatomical specificity, and future studies incorporating localized delivery approaches (e.g., stereotaxic cannula-based infusion) and real-time recording techniques (e.g., fiber photometry or miniature microscopy) will be essential to enhance spatial and temporal resolution.

In summary, our study demonstrates the essential role of astrocytes in stress-induced anxiety sensitivity via the BDNF and IFN signaling pathways. These findings advance our understanding of astrocyte–neuron coordinated regulation in anxiety pathophysiology and highlight astrocytic BDNF as a potential therapeutic target for anxiety disorders.

## Materials and Methods

### Animals

Experiments were conducted using male mice aged 8 weeks unless otherwise specified. All procedures involving mice were approved by the Institutional Animal Care and Use Committee of Southern Medical University (Guangzhou, China). Mice were group housed (3 to 5/cage) under a 12-h light/dark cycle (lights on at 8:00 AM and lights off at 8:00 PM), with ad libitum access to food and water. Experimenters blinded to treatment conducted all experiments and data analyses. C57BL/6J mice were provided by the Experimental Animal Center at Southern Medical University.

### RS model

Mice were individually positioned into a well-ventilated polypropylene conical tube (50 ml) for 2 h daily at the same time, over a course of 3, 10, or 21 days (with 3 days for subthreshold and 10 days for conventional CRS). After each session, mice were returned to their home cages with ad libitum access to food and water.

### Drug administration

To investigate the modulation of BDNF/TrkB signaling, mice were administered either the TrkB agonist, 7,8-DHF (Sigma-Aldrich, #D5446), or the antagonist, ANA-12 (Selleck Chemicals, #S7745). Both compounds were dissolved in saline with a minimal amount of dimethyl sulfoxide (DMSO) to enhance solubility, with 7,8-DHF administered at 10 mg/kg and ANA-12 at 0.5 mg/kg. In chronic treatment protocols, these drugs were administered i.p. once daily following RS modeling. For acute administration experiments, 7,8-DHF was injected i.p. 30 min before behavioral testing.

To stimulate IFN signaling, tilorone dihydrochloride (Bidepharm, #BD154145) was administered (30 mg/kg i.p.) daily after RS modeling.

For chemogenetic manipulations, clozapine-N-oxide (CNO; 5 mg/kg; MCE, #HY-17366) was freshly dissolved in saline (0.9% NaCl) and injected i.p. 30 min before experiments.

For each experiment, all drugs were prepared fresh on the day. Control mice were administered an equivalent volume of vehicle (saline with DMSO).

### Stereotaxic injections

After being anesthetized with pentobarbital sodium (75 mg/kg, i.p.), the mice were positioned on a stereotaxic frame (RWD) with body temperature maintained throughout the procedure. Ophthalmic ointment was applied to prevent corneal drying. The scalp was sterilized and incised, exposing the skull for craniotomies at the dorsal and ventral hippocampal CA1 coordinates, relative to bregma: dorsal hippocampus: anterior–posterior (AP): −2.00 mm, medial–lateral (ML): ± 1.75 mm, dorsal–ventral (DV): −1.60 mm; ventral hippocampus: AP: −3.20 mm, ML: ±3.20 mm, DV: −3.00 mm. Using a 5-μl microsyringe (Gaoge), viral solution was infused at a slow rate (0.1 μl/min) for a total of 0.3 μl. To minimize backflow, the needle remained in place for 5 min postinjection before being slowly withdrawn. Following suturing, mice were placed in a heated recovery chamber before returning to their home cages. Experiments were conducted at least 4 weeks following viral injection.

The viral constructs included AAV2/9-GFAP-shRNA (BDNF)-eGFP for BDNF knockdown and AAV2/9-GFAP-eGFP as a control, AAV2/9-CaMK2α-hM4Di-mCherry for DREADD-based inhibition and AAV2/9-CaMK2α-mCherry as its control, and AAV2/5-GfaABC_1_D-GCaMP6f and AAV2/5-GfaABC_1_D-BFP-hPMCA2w/b. All viruses were produced by Sunbio Medical Biotechnology (Shanghai) or BrainVTA Viral Biotechnology (Wuhan) Company.

### Behavioral experiments

All behavioral tests were carried out with male mice and their littermates. To minimize stress, mice were handled by investigators for 3 consecutive days and were placed in the experimental room for at least 30 min for acclimatization.

### Open field test

The OFT was used to assess anxiety-like behaviors and general locomotor activity using the VersaMax Animal Activity Monitoring System (Accuscan Instruments). Mice were gently positioned in the center of a white opaque plastic chamber (40 × 40 × 30 cm) and allowed to move freely for 5 min. Parameters such as total distance and central time were automatically calculated using VersaDat software. The center zone was the inner 20 × 20 cm area. Decreased central zone exploration was interpreted as indicators of anxiety-like behaviors. To eliminate olfactory cues, the chamber was cleaned with 70% ethanol between each trial.

### Elevated plus maze

The EPM was conducted to further evaluate anxiety-like behaviors. The apparatus was made up of 2 opposing closed arms (30 × 5 × 15 cm) and open arms (30 × 5 × 0.5 cm) emanating from a common central platform (5 × 5 cm) in a plus shape (50 cm above floor level). Mice were positioned in a central platform and allowed to explore for 5 min. An overhead video camera was used to record their behavior, and parameters, including time spent in each arm and distance moved, were automatically analyzed using EthoVision XT 11.5 software (Noldus). A decrease in open-arm exploration was considered indicative of anxiety-like behaviors. The apparatus was cleaned with 70% ethanol between tests to eliminate scent cues.

To assess neuronal activation, mice were returned to a quiet room for 90 min immediately following the EPM experiment. Subsequently, animals were sacrificed, and the brains were collected for c-Fos immunohistochemistry.

### Sucrose preference test

The SPT was performed to assess anhedonia. Mice were habituated with 2 bottles containing a 1% sucrose solution for 24 h. Following this habituation period, mice underwent 24 h of water restriction. During the 24-h test period, mice had access to one bottle with 1% sucrose solution (w/v) and one bottle with water. Bottle positions were alternated every 6 h to control for side preference, and fluid intake was recorded. The sucrose preference is measured as the percentage of total liquid intake that consisted of sucrose solution. Reduced sucrose preference was interpreted as indicative of anhedonia.

### Forced swim test

The FST evaluated behavioral despair. Mice were individually placed into a transparent cylinder (with 20 cm diameter and 20 cm height) filled with water (depth of water: 15 cm and temperature: 23 to 25 °C). Mice were forced to swim for 6 min, with recording in the last 4 min. Immobility time, defined as duration spent floating with minimal movement to keep the head above water, was measured as an indicator of behavioral despair, a core symptom of depression. After each session, mice were immediately dried and put back into their home cages. The water was replaced between trials.

### Fiber photometry

Following the injection of AAV2/5-GfaABC_1_D-GCaMP6f alone or in combination with AAV2/5-GfaABC_1_D-BFP-hPMCA2w/b into the ventral hippocampal CA1 region (AP: −3.20 mm, ML: ±3.20 mm, DV: −3.00 mm), an optical fiber (outer diameter [OD] of 200 μm, numerical aperture [NA] of 0.37, purchased from Thinker Tech) was implanted on the same site. After a 2-week recovery, GCaMP6f fluorescence signals were recorded using a fiber photometry system (Thinker Tech) with a 488-nm excitation (10 to 15 μW) during RS exposure. Stimulus trials were separated by randomized inter-trial intervals (1 to 3 min). Calcium transients were quantified as Δ*F*/*F* (%) = [(*F − F*_0_)/*F*_0_] × 100%, where *F*_0_ is defined as the average fluorescence over the 10-s prestimulus baseline. Data were analyzed using custom MATLAB scripts.

### Immunohistochemistry

After being deeply anesthetized, the mice were perfused intracardially with ice-cold phosphate-buffered saline (PBS), followed by 4% paraformaldehyde (PFA) in PBS. Brains were extracted, postfixed in 4% PFA overnight at 4 °C, and equilibrated in 30% sucrose solution until they sank. Using a microtome-cryostat (Leica CM1950), coronal brain sections (40 μm) were cut at intervals of 240 μm through the hippocampus (dorsal: bregma −1.06 to −2.54 mm; ventral: −2.54 to −3.80 mm), medial prefrontal cortex (every fourth section from bregma, 1.98 to 1.34 mm), nucleus accumbens (every fourth section from bregma, 1.78 to 0.86 mm), amygdala (every fourth section from bregma, −0.82 to −2.06 mm), and dorsal raphe nucleus (every fourth section from bregma, −4.36 to −4.84 mm). Floating sections were washed 3 times in PBS (10-min intervals) and exposed to 10 mM sodium citrate buffer at 80 °C for 30 min. After cooling down to room temperature, sections were incubated in blocking solution (PBS containing 1% Triton X-100 and 5% bovine serum albumin) for 2 h. The sections were incubated with the following primary antibodies at 4 °C overnight: c-Fos (1:500, Millipore, #ABE457), GFAP (1:500, CST, #80788), IFN-β (1:500, Affinity Biosciences, #DF6471), IFN-γ (1:500, Proteintech, #15365-1-AP), and GFP (1:500, Invitrogen, #G10362). After being washed, the sections were incubated for 2 h at room temperature with Alexa Fluor-conjugated secondary antibodies (1:500, Invitrogen). 4′,6-Diamidino-2-phenylindole (DAPI; 1 μl/ml, Sigma, #D9542) was used to counterstain the nuclei, and coverslips were mounted with antifade mounting medium. Fluorescent images were obtained using a confocal microscope (Nikon A1R or Zeiss LSM 900).

### Quantitative cell counting

C-Fos^+^ cells and GFAP^+^ astrocytes were quantified in immunofluorescence-stained hippocampal sections using the Cell Counter plugin in ImageJ. Confocal images (20×) were analyzed by a blinded investigator, with cells identified based on fluorescence intensity and nuclear colocalization (DAPI^+^).

### Astrocyte morphology analysis

Astrocytes were sparsely labeled by stereotaxic injection of low-titer AAV-GFAP-eGFP (10^9^ to 10^10^ vg/ml, 0.3 μl). High-resolution z-stacks (1-μm intervals) were acquired using a 60×/1.42 NA oil-immersion objective and reconstructed in 3-dimensional (3D) using Imaris v9.0.0 (Bitplane). The “*Filaments*” module was employed to quantify total process length and perform Sholl analysis, with concentric spheres generated at 3-μm radial increments from the soma centroid. Only isolated astrocytes with fully intact arborization were included. Morphometric parameters were derived from at least 10 cells per group, with quantification performed by investigators blinded to experimental conditions.

### Real-time quantitative polymerase chain reaction

Using TRIzol reagent (Invitrogen, #15596018), RNA was extracted and quantified with a NanoDrop spectrophotometer (Thermo). cDNA was reverse-transcribed from 1 μg of RNA using a qPCR RT Kit (TOYOBO, #FSQ-101) according to the manufacturer’s instruction. Real-time quantitative polymerase chain reaction (RT-qPCR) was carried out using SYBR Premix Ex Taq II (Takara, #RR420A) on an ABI Prism 7500 Fast sequence detection system (Applied Biosystems). The cycling protocol consisted of an initial step at 95 °C for 3 min, followed by 40 cycles as follows: denaturation at 95 °C for 15 s, annealing at 60 °C for 30 s, and extension at 72 °C for 30 s. Primer sequences were as follows:

*Bdnf*: forward: AGGATCCCCATCACAATCTTACA; reverse: GCCACTGACCACACAATTGCT.

*Ntrk2*: forward: CTGGGGCTTATGCCTGCTG; reverse: AGGCTCAGTACACCAAATCCTA.

*Gapdh*: forward: TCACCACCATGGAGAAGGC; reverse: GCTAAGCAGTTGGTGGTGCA.

Relative target gene expression was calculated using the ΔΔCT method, with *Gapdh* as the internal control.

### Western blotting

Tissues were homogenized in ice-cold radioimmunoprecipitation assay buffer (Beyotime) and centrifuged at 12,000 rpm for 10 min at 4 °C to collect the supernatant. The BCA protein assay kit (Thermo, #23227) was used to measure the protein concentration. Protein samples (20 to 30 μg) were mixed with 6× SDS loading buffer, boiled for 10 min, separated by electrophoresis, and transferred onto PVDF membranes (Millipore). After blocking with 5% nonfat milk in TBST for 1 hour at room temperature, the membranes were incubated overnight at 4 °C with the following antibodies: BDNF (1:1,000, Abcam, #ab108319), TrkB (1:1,000, Proteintech, #13129-1-AP), IFN-β (1:1,000, Affinity Biosciences, #DF6471), IFN-γ (1:1,000, Affinity Biosciences, #DF6045), and GAPDH (1:5,000, Proteintech, #60004-1-Ig). After TBST washes, membranes were incubated with HRP-conjugated secondary antibodies (anti-rabbit IgG-HRP, 1:50,000, Proteintech, #SA00001-2; anti-mouse IgG-HRP, 1:50,000, Proteintech, #SA00001-1) at room temperature for 2 h. Using an ECL detection reagent (Thermo), the protein bands were visualized and imaged with a ChemiDoc Imaging System (Bio-Rad). Densitometry was analyzed using ImageJ software.

### Transcriptome sequencing

Hippocampal CA1 tissue was microdissected and flash-frozen in liquid nitrogen. Total RNA was isolated, and its quality and concentration were measured. Only samples with an RNA integrity number (RIN) greater than 7.0 were included. Using the Illumina TruSeq Stranded mRNA Kit, libraries were prepared and sequenced on an Illumina NovaSeq 6000 platform. The sequencing generated paired-end 150-bp reads, aiming for a depth of 20 million reads per sample. Quality control for raw reads was performed with FastQC, and Cutadapt was used to trim adapters. The alignment of reads to the mouse reference genome (GRCm38) was done using HISAT2 (v2.2.1), and transcript quantification was performed with HTSeq (v0.6.1). Using DESeq2 in R, differential gene expression analysis was performed, with significance criteria set to an adjusted *P* value of <0.05 and a |Fold Change| >1.5. Enrichment analyses, including GO, KEGG pathways, and GSEA, were conducted using the “ClusterProfiler” package in R.

### Slice preparation and electrophysiological recording

Hippocampal slice preparations were performed as previously described [13,56]. After being deeply anesthetized, mice were perfused intracardially with ice-cold, high-sucrose artificial cerebrospinal fluid (ACSF). This high-sucrose ACSF contained 220 mM sucrose, 26 mM NaHCO_3_, 10 mM D-glucose, 12 mM MgSO_4_, 2 mM KCl, 1.3 mM NaH_2_PO_4_, and 0.2 mM CaCl_2_, equilibrated with 95% O_2_/5% CO_2_. The mouse brains were quickly removed and cooled in oxygenated high-sucrose ACSF. Using a vibratome (Leica VT1000S), coronal slices (300 μm thick) containing the hippocampus were cut and moved to an incubation chamber with oxygenated ACSF at 34 °C for 30 min. Before recording, the slices were stored at room temperature for at least 1 h. The Standard ACSF contained 124 mM NaCl, 3 mM KCl, 1.25 mM NaH_2_PO_4_, 2 mM CaCl_2_, 26 mM NaHCO_3_, and 10 mM D-glucose, continuously bubbled with 95% O_2_/5% CO_2_ (pH = 7.4, 300 to 310 mOsm).

For the recording process, the slices were placed into a recording chamber and perfused with oxygenated ACSF (constant rate of 2 ml/min), while the temperature was maintained at 30 ± 1 °C. Patch pipettes were pulled on a horizontal puller (P97, Sutter Instruments) from filamented borosilicate glass capillaries. Whole-cell patch-clamp recording from CA1 neurons was visualized with an upright microscope equipped with an infrared-sensitive camera (DAGE-MTI, IR-1000E) and a 40× water-immersion lens (Nikon, ECLIPSE FN1). Electrophysiological data were obtained with a Multiclamp 700B amplifier (Molecular Devices), online-filtered at 2 kHz, and digitized at 10 kHz using a Digidata 1440A (Molecular Devices).

Whole-cell recordings were obtained from hippocampal CA1 pyramidal neurons within a coronal range from approximately anterior–posterior = −1.7 to −3.4 mm, without subregional selection bias. To measure sEPSCs and sIPSCs in the same neurons, voltage-clamp recordings were conducted at the reversal potential for GABA_A_ receptor-mediated EPSCs (−60 mV) and at the reversal potential for ionotropic glutamate receptor-mediated IPSCs (0 mV). The pipette resistance was typically 6 to 8 MΩ after being filled with an internal solution: 110 mM Cs_2_SO_4_, 2 mM MgCl_2_, 0.5 mM CaCl_2_, 5 mM HEPES, 5 mM TEA, 5 mM EGTA, and 5 mM Mg-ATP (pH = 7.3, 285 mOsm). To evaluate the PPR, paired stimuli were delivered through a bipolar electrode positioned in Schaffer collateral (SC)-CA1 pathway, and the PPR was calculated as the amplitude ratio of the second EPSC to that of the first EPSC. To determine AMPA and NMDA current components, EPSCs were recorded at holding potentials of −70 mV (for AMPA) and +40 mV (for NMDA). AMPA currents were measured as the peak current within the first 5 ms after stimulation, while NMDA currents were quantified as the amplitude 50 ms poststimulation (averaging across 10 sweeps). The AMPA/NMDA ratio was calculated by dividing the AMPA current by the NMDA current. The addition of bicuculline (BMI, 20 μM) to the bath solution was used to block GABA_A_ receptor-mediated currents, allowing for the isolation of glutamate receptor-mediated EPSCs.

For chemogenetic manipulations, CNO (5 μM) was bath-applied to brain slices. Action potential (AP) was recorded in current-clamp mode. The patch pipettes were filled with a K^+^-gluconate internal recording solution, which contained 130 mM K-gluconate, 10 mM HEPES, 20 mM KCl, 0.2 mM EGTA, 10 mM Na-creatine, 4 mM Mg-ATP, and 0.3 mM Na-GTP (pH = 7.3, 285 mOsm). Neuronal spike activity was recorded for a baseline period from −2 to 0 min before CNO application, and for a postapplication period from 3 to 5 min after CNO was added.

For each cell, recordings commenced 2 to 5 min post-break-in after stabilization of the holding potential. Cells with a series resistance of less than 30 MΩ, an absolute leak current of less than 100 pA, and stable throughout the experiment were included. Synaptic currents were analyzed using Minianalysis, and other data were analyzed using Clampfit 10.7.

### Statistical analysis

Statistical analyses were performed using GraphPad Prism 7. Results are presented as mean ± standard error. Sample sizes for each experiment were determined according to prior research to guarantee adequate power for detecting statistical differences. Normality was assessed with the Shapiro–Wilk test. When comparing 2 groups, unpaired *t* tests or Mann–Whitney *U* tests were employed. For comparisons among multiple groups, 1-way or 2-way analysis of variance (ANOVA) was used, with Bonferroni post hoc tests employed to assess group differences. Significance levels were defined as *P < 0.05, **P < 0.01, ***P < 0.001, or ****P < 0.0001.

## Data Availability

Data supporting the findings of this study may be accessed from the corresponding authors upon reasonable request.
