# Supplementary material for: Astrocytic BDNF Modulates Sensitivity to Stress-Induced Anxiety-Like Behaviors
Source: Research (Wash D C). 2025 Aug 7;8:0818. doi: 10.34133/research.0818 (PMC12329215; doi:10.34133/research.0818)
Supplement: Supplementary 1 — Figs. S1 to S8 [file research.0818.f1.docx]

**Supporting Information**

Title: Astrocytic BDNF Modulates Sensitivity to Stress-Induced Anxiety-Like Behaviors

**Author:** Wei-Peng Li^†,1,2,6^, Gui-Yu Liu^†,2,6^, Shi-Yun Wang^2,6^, Jiao Hu^2,6^, Wen-Juan Ji^2,6^, Jun-Ming Zhu^2,6^, Qing-Yu Chang^2^, Tian-Yi Li^2^, Guo-Rong Wei^1,6^, Jian-Qing Shang^1,6^, Hong-Zhan Li^1,6^, Fu-Hua Peng^4^, Yun-Yan Zhao^*,5^, Xiao-Hong Su^*,4^, Wei Xie^*,#,1,2,3,6^


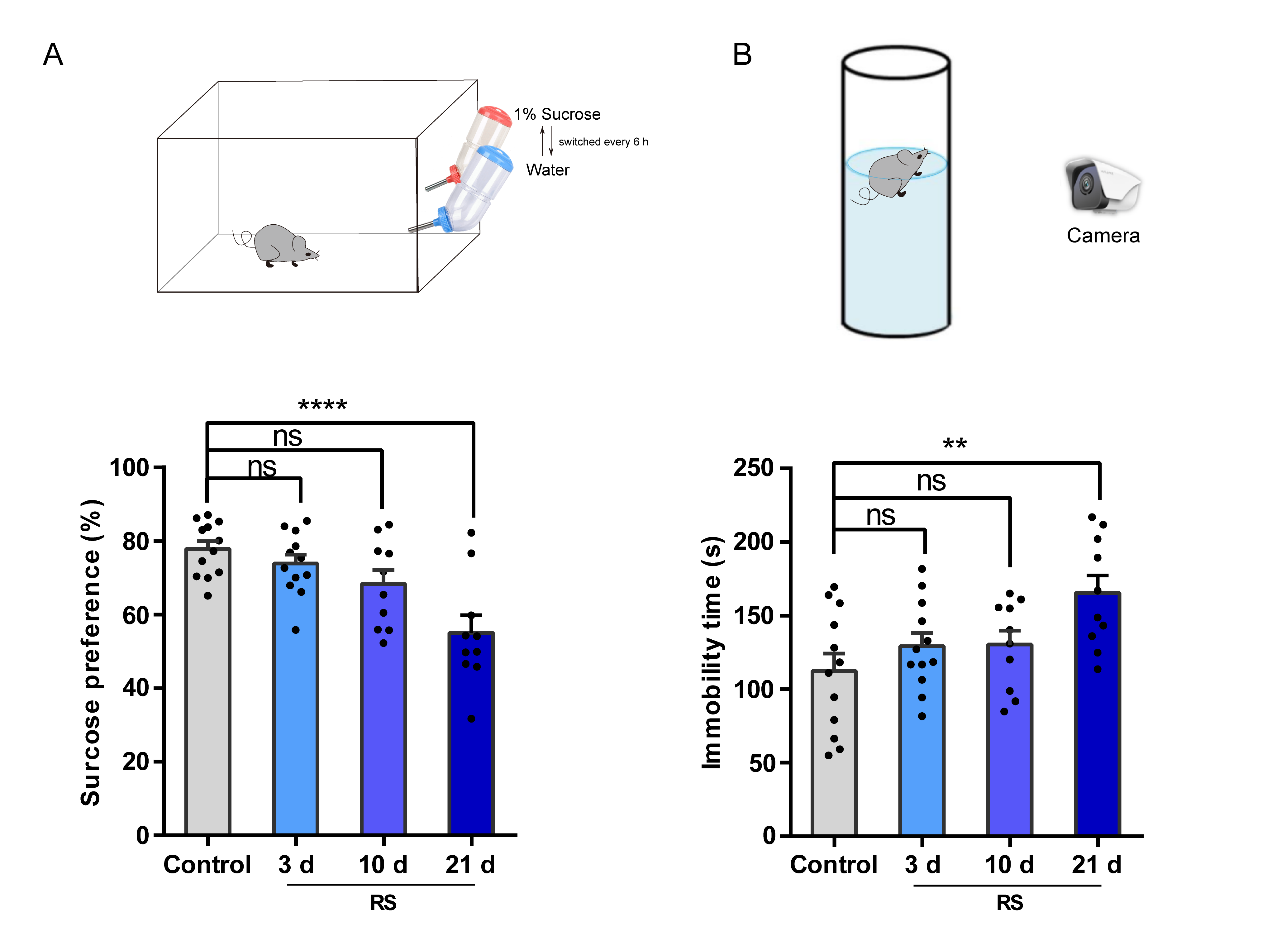


**Supplementary Fig. 1. Restraint stress alters depressive-like behaviors in mice**

(A) SPT schematic diagram and sucrose consumption (control, n = 12; 3d-RS, n = 12; 10d-RS, n = 10; 21d-RS, n = 10; one way ANOVA; F _(3, 40)_ = 9.012, P = 0.0001).

(B) FST schematic diagram and the immobility time (control, n = 12; 3d-RS, n = 12; 10d-RS, n = 10; 21d-RS, n = 10; one way ANOVA; F _(3, 40)_ = 4.239, P = 0.0108).


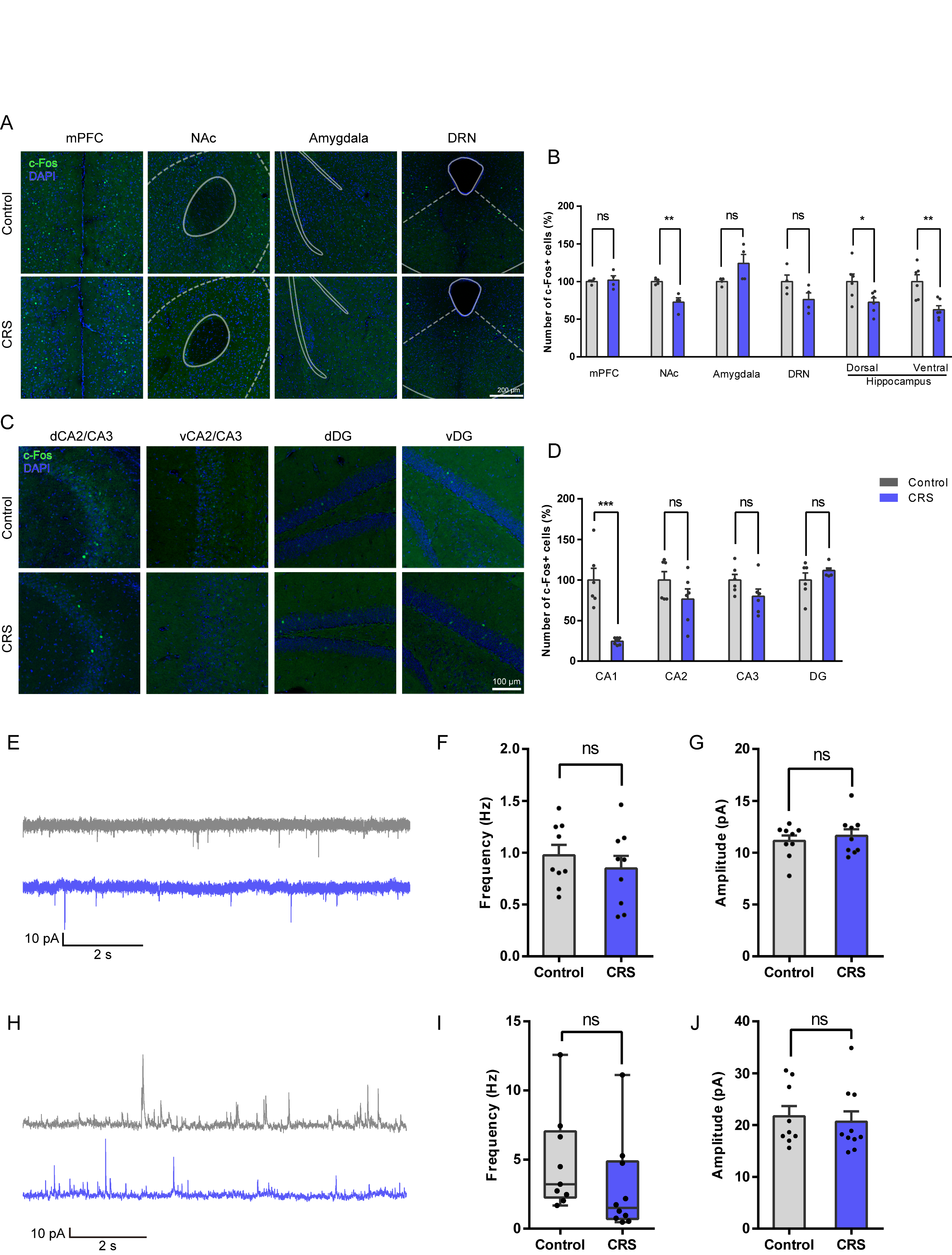


**Supplementary Fig. 2. CRS selectively alters neuronal activity and synaptic transmission in stress-related brain regions**

(A-B) Representative images and quantification of c-Fos+ cells in the medial prefrontal cortex (mPFC), nucleus accumbens (NAc), amygdala, dorsal raphe nucleus (DRN) and hippocampus of control and CRS-treated mice (mPFC, NAc, amygdala and DRN: n = 4 per group; hippocampus: n = 6 per group; unpaired two-tailed Student’s t test; mPFC, t_6_ = 0.3103, P = 0.7668; NAc, t_6_ = 4.431, P = 0.0044; amygdala, t_6_ = 1.958, P = 0.0979; DRN, t_6_ = 1.944, P = 0.1000; dorsal hippocampus, t_10_ = 2.386, P = 0.0382; ventral hippocampus, t_10_ = 3.615, P = 0.0047). Scale bar, 200 μm.

(C-D) Representative images and quantification of c-Fos+ cells in the hippocampal subregions of control and CRS-treated mice (n = 6 per group; unpaired two-tailed Student’s t test; CA1, t_10_ = 5.229, P = 0.0004; CA2, t_10_ = 1.445, P = 0.1790; CA3, t_10_ = 1.759, P = 0.1090; DG, t_10_ = 1.246, P = 0.2411). Scale bar, 100 μm.

(E) Example traces of 10 s recordings of sEPSCs. Scale bars, 10 pA, 2 s.

(F-G) Frequency and amplitude of sEPSCs recorded from dentate granule cells (DGCs) in control and CRS mice (n = 3 mice per group, average of 2-4 cells from each mouse; unpaired two-tailed Student’s t test; frequency, t_16_ = 0.804, P = 0.4332; amplitude, t_16_ = 0.6014, P = 0.556).

(H) Example traces of 10 s recordings of sIPSCs. Scale bars, 10 pA, 2 s.

(I-J) Frequency and amplitude of sIPSCs recorded from DGCs in control and CRS mice (n = 3 mice per group, average of 2-4 cells from each mouse; frequency, Mann Whitney test, Z = -1.639, P = 0.0786; amplitude, unpaired two-tailed Student’s t test, t_17_ = 0.3805, P = 0.7083).


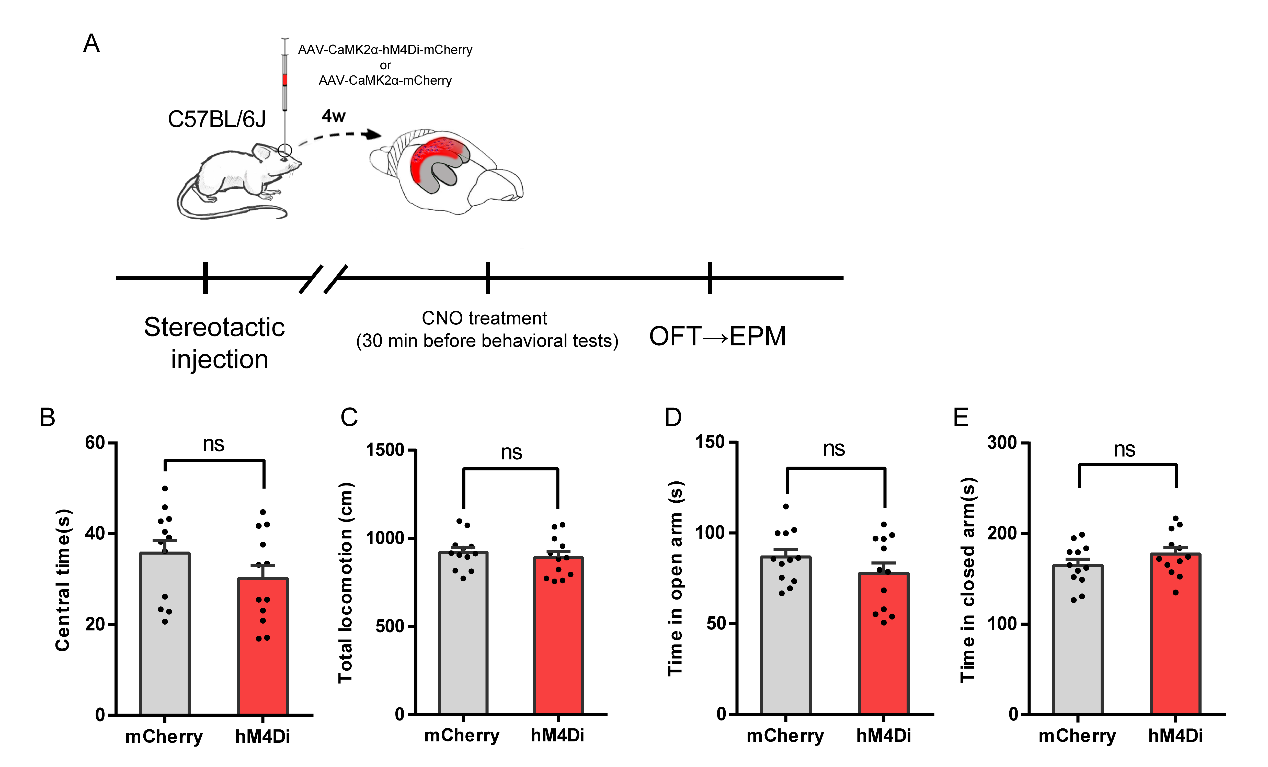


**Supplementary Fig. 3. Selective silencing of CA1 pyramidal neurons does not induce anxiety-like behaviors**

(A) Schematic of chemogenetic inhibition of CA1 pyramidal neurons.

(B-C) Center time and locomotion in OFT (n = 12 per group; unpaired two-tailed Student’s t test; central time, t_22_ = 1.376, P = 0.1828; locomotion, t_22_ = 0.7015, P = 0.4903).

(D-E) Open and closed arm time in EPM (n = 12 per group; unpaired two-tailed Student’s t test; open arm, t_22_ = 1.1.264, P = 0.2195; closed arm, t_22_ = 1.276, P = 0.2151).


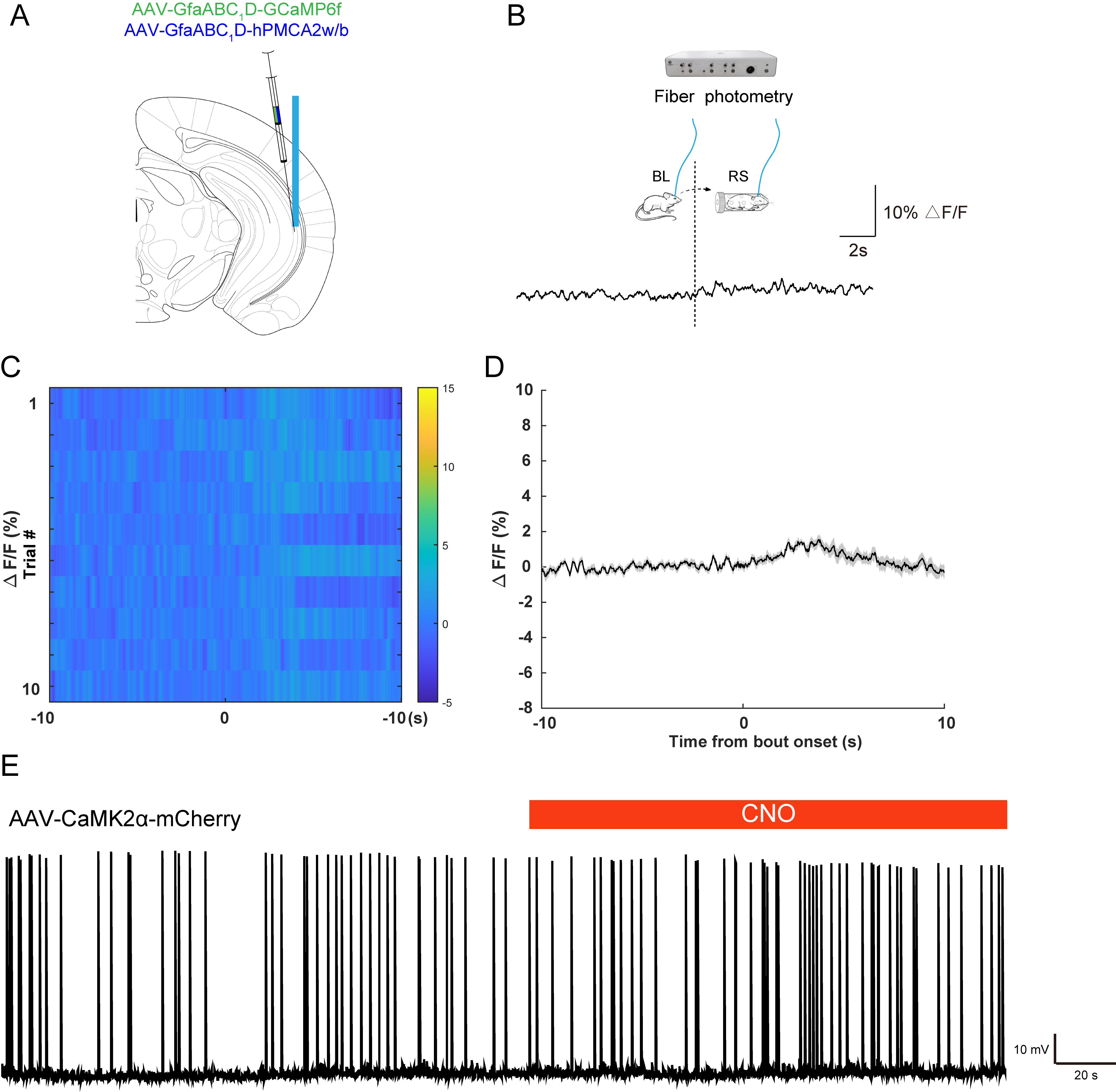


**Supplementary Fig. 4. Validation of hPMCA2w/b-mediated inhibition of astrocytic Ca^2+^ signals and chemogenetic control AAV vector.**

(A) Schematic illustrating AAV-GfaABC_1_D-GCaMP6f and AAV-GfaABC_1_D-hPMCA2w/b into ventral CA1, along with optical fiber implantation.

(B) Representative Ca^2+^ signal trace in CA1 astrocytes of hPMCA2w/b-expressing mice in response to restraint stress (RS). Scale bars, 10% ΔF/F, 2 s.

(C-D) Representative heatmaps and averaged Ca^2+^ transients evoked by RS stimulation in hPMCA2w/b-treated mice.

(E) Representative current-clamp recording of AAV-CaMK2α-mCherry-infected CA1 pyramidal neuron with CNO application. Scale bars, 10 mV, 20 s.


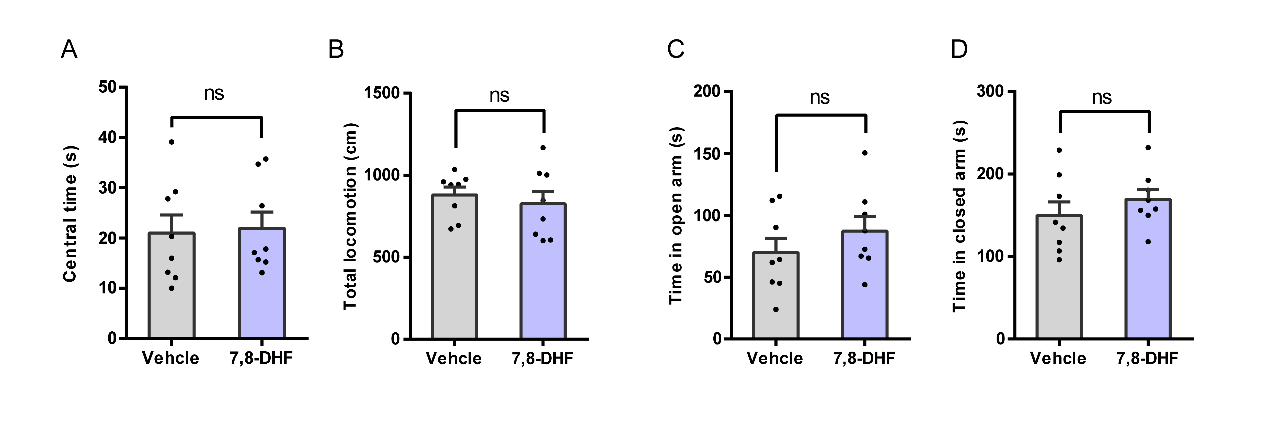


**Supplementary Fig. 5. Acute 7,8-DHF administration does not affect anxiety-like behaviors**

(A-B) Time in center and total locomotion in the OFT (n = 8 per group; unpaired two-tailed Student’s t test; central time, t_14_ = 0.2073, P = 0.8387; locomotion, t_14_ = 0.5899, P = 0.5646).

(C-D) Time in open and closed arm in the EPM (n = 8 per group; unpaired two-tailed Student’s t test; open arm, t_14_ = 1.054, P = 0.3098; closed arm, t_14_ = 0.967, P = 0.3499).


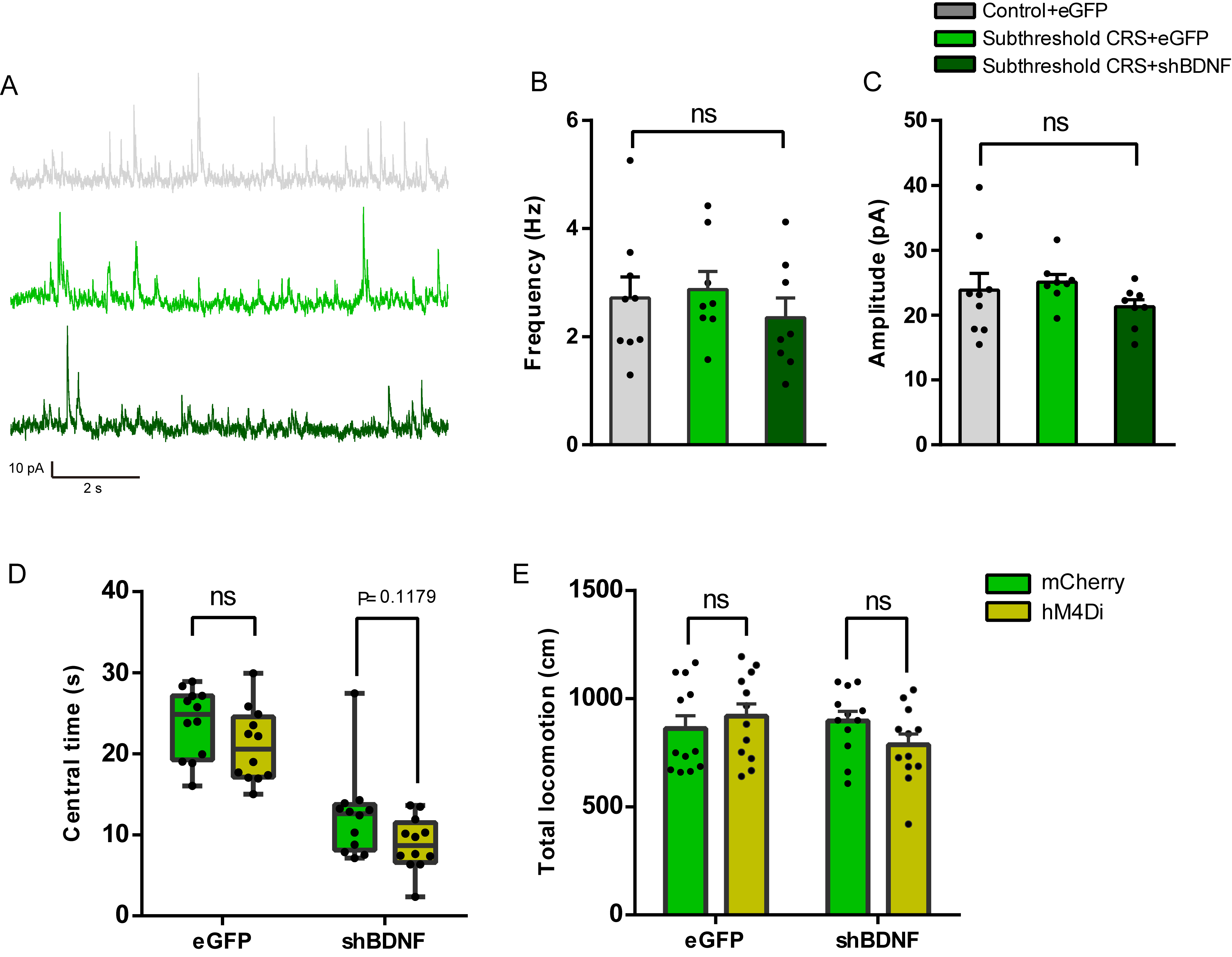


**Supplementary Fig. 6. Astrocytic BDNF knockdown does not alter sIPSCs or anxiety-like behaviors in OFT under subthreshold CRS**

(A) Example traces of 10 s recordings of sIPSCs from CA1 pyramidal neurons in shBDNF-eGFP and control eGFP with subthreshold CRS. Scale bars, 10 pA, 2 s.

(B-C) Frequency and amplitude of sIPSCs (n = 3 mice per group, average of 2-4 cells from each mouse; one-way ANOVA; frequency, F _(2, 22)_ = 0.5047, P = 0.6105; amplitude, F _(2, 22)_ = 1.087, P = 0.3546).

(D-E) OFT central time and total locomotion in hM4Di-treated AAV-GFAP-shBDNF-eGFP mice after 3-day subthreshold CRS (n=12 per group; two-way ANOVA; central time, F _(1, 44)_ = 6.053, P = 0.0179; locomotion, F _(1, 44)_ = 0.2673, P = 0.6077).


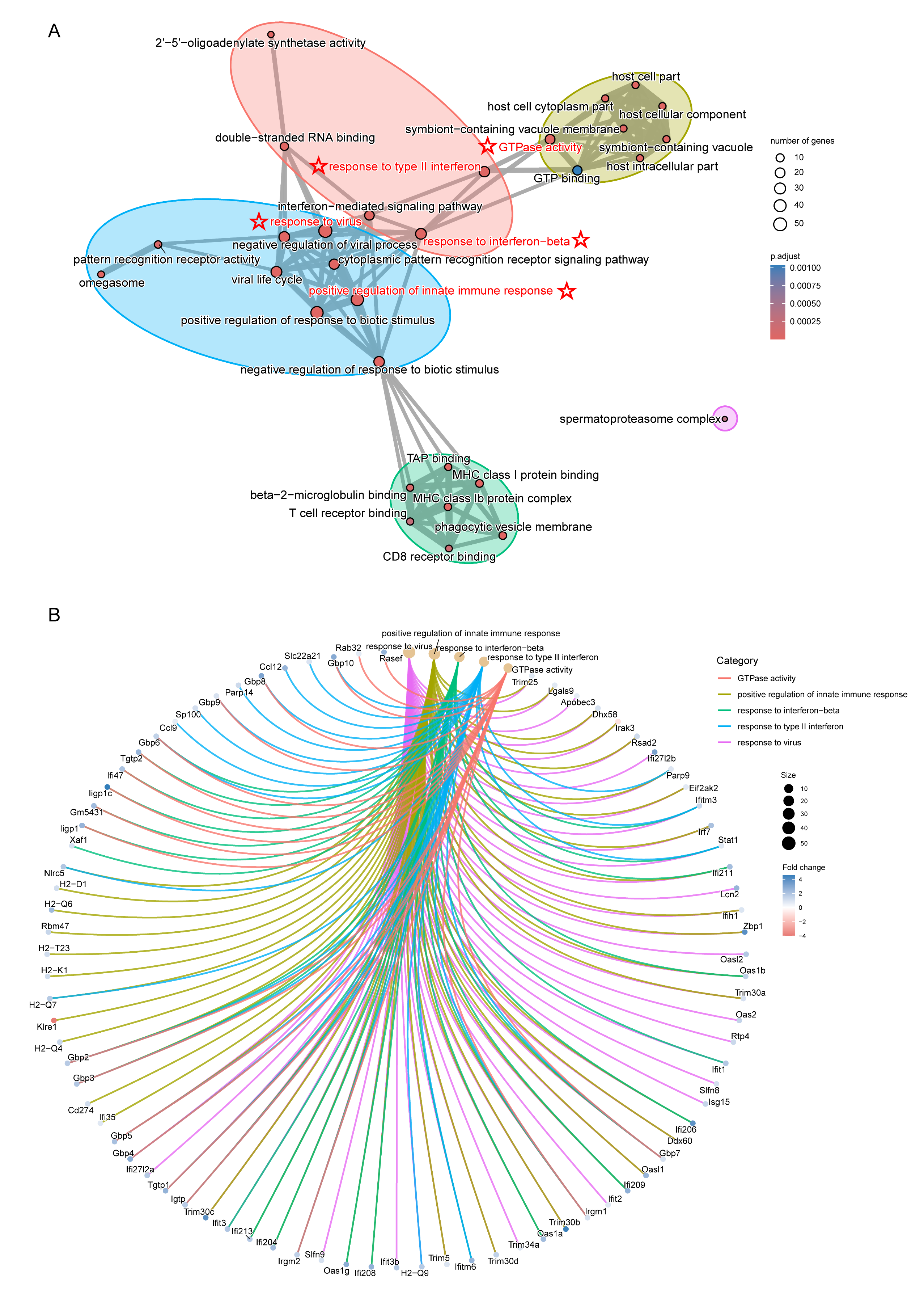


**Supplementary Fig. 7. GO term enrichment analysis via enrichment map and Cnetplot**

(A) Functional enrichment map of top GO terms.

(B) Cnetplot showing gene names associated with the IFN-related sipathway.


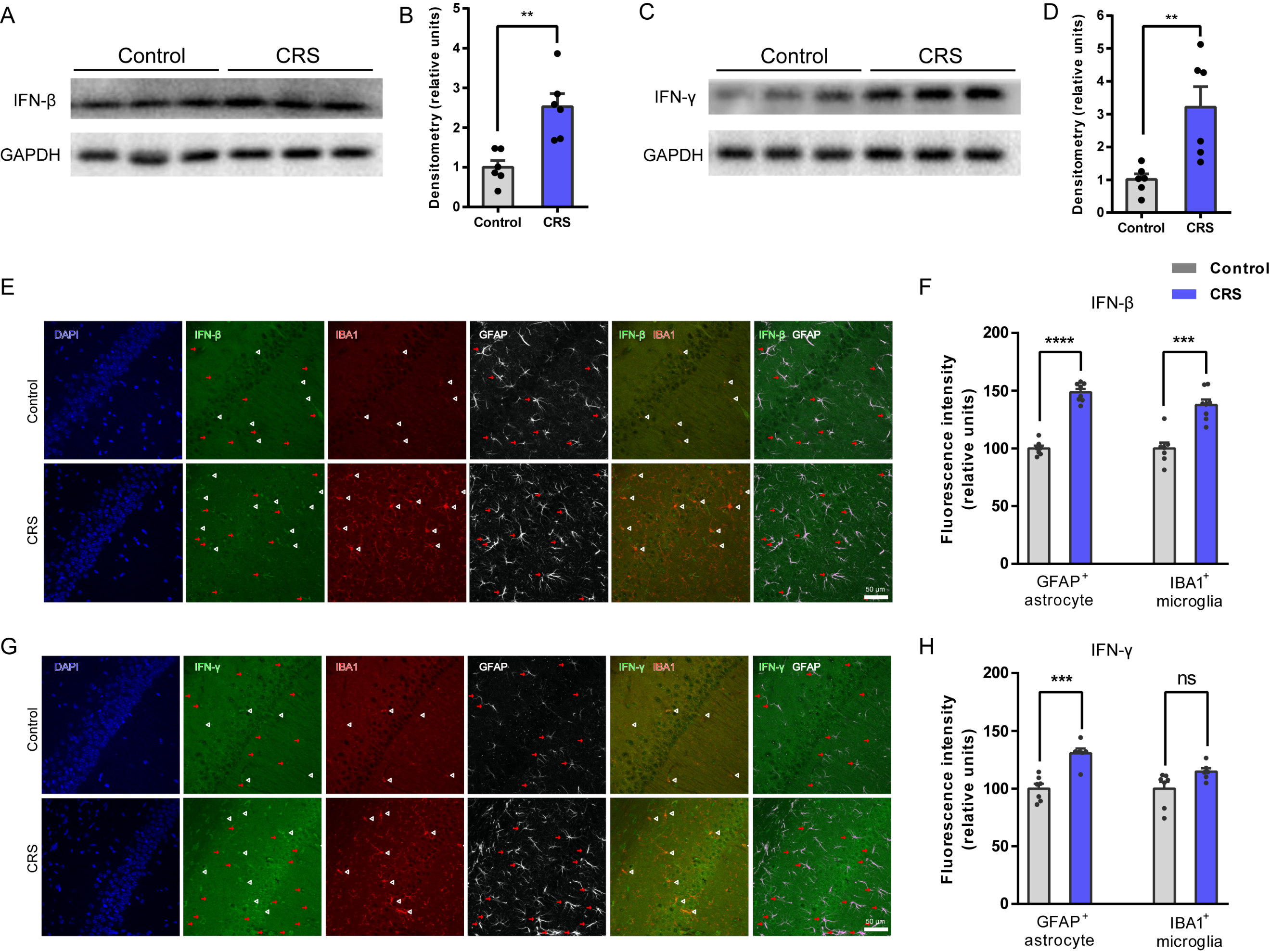


**Supplementary Fig. 8.** **CRS elevates IFN-β and IFN-γ expression and enhances their immunoreactivity in hippocampal astrocytes and microglia**

(A-B) Western blotting analysis of IFN-β protein in control and CRS-treated mice (n = 6 mice per group; unpaired two-tailed Student’s t test; IFN-β, t_10_ = 3.4, P = 0.0068).

(C-D) Western blotting analysis of IFN-γ protein in control and CRS-treated mice (n = 6 mice per group; unpaired two-tailed Student’s t test; IFN-γ, t_10_ = 4.108, P = 0.0021).

(E-F) Representative images and quantification of IFN-β immunoreactivity (green) in IBA1^+^ microglia (red) and GFAP^+^ astrocytes (white) in the CA1 region of control and CRS mice (n = 35 cells from 7 slices, 3 control mice, n = 40 cells from 8 slices, 3 CRS; unpaired two-tailed Student’s t test; GFAP^+^ astrocytes, t_13_ = 13.04, P < 0.0001; IBA1^+^ microglia, t_13_ = 5.447, P = 0.0001). Red arrows indicate astrocytes, and white triangles denote microglia. Scale bar, 50 μm.

(G-H) Representative images and quantification of IFN-γ immunoreactivity (green) in IBA1^+^ microglia (red) and GFAP^+^ astrocytes (white) in the CA1 region of control and CRS mice (n = 35 cells from 7 slices, 3 control mice, n = 30 cells from 6 slices, 3 CRS mice; unpaired two-tailed Student’s t test; GFAP^+^ astrocytes, t_11_ = 5.236, P = 0.0003; IBA1^+^ microglia, t_11_ = 2.172, P = 0.0526). Red arrows indicate astrocytes, and white triangles denote microglia. Scale bar, 50 μm.
